# Supplementary material for: A Unified View of Topological Phase Transition in Band Theory
Source: Research (Wash D C). 2020 May 23;2020:7832610. doi: 10.34133/2020/7832610 (PMC7262673; doi:10.34133/2020/7832610)
Supplement: Supplementary Materials — General band evolution; details of methodology; TPTs of two-dimensional crystals and quasicrystals; random vacancy; thermal fluctuation; validity of the linear scaling for different forms of electron hopping. Figure S1: schematic illustration of topological phase transition. Figure S2: the calculation of formation of band structure from discrete levels of isolated atoms by decreasing average bong length. Figure S3: five 2D Bravais lattices. Figure S4: the eight lattices based on semiregular Archimedean tilings. Figure S5: several decorated trigonal lattices. Figure S6: atomic model of the Penrose-type and the Ammann-Beenker-type quasicrystal lattices. Figure S7: the linear scaling relation in various 2D crystalline lattices using the power-law decay function (1/rij2). Figure S8: the linear scaling relation in various 2D crystalline lattices using the exponentially decay function (e−2.3(rij − 1)). Table S1: the electron hopping potential γ and the critical bond length Lc for TPT in typical 2D crystals and quasicrystal lattices. [file 7832610.f1.pdf]

Supplementary Material for "A Unified View of Topological Phase Transition in Band Theory"

Huaqing Huang[1,2] and Feng Liu[a1]

[1] *Department of Materials Science and Engineering, University of Utah, Salt Lake City, Utah 84112, USA*

[2] *School of Physics, Peking University, Beijing 100871, China*

(Dated: January 30, 2020)

[a] Corresponding author: fliu@eng.utah.edu

CONTENTS

General band evolution 2

Details of methodology 2
Tight-binding model 2
Calculation of topological invariants 4

TPTs of two-dimensional crystals and quasicrystals 5

Random vacancy 6

Thermal fluctuation 6

Validity of the linear scaling for different form of electron hoppings 7

References 7

GENERAL BAND EVOLUTION

One of the most simple and straightforward way to understand the band structure is to conceptually constructing the solid from isolated atoms, as illustrated in textbooks of solid state physics [1, 2].The origin of band structure can be qualitatively explained by starting with the discrete orbital levels of free atoms and interpreting the bands by spreading the atomic levels through interactions in the solid. In this interpretation every band in the band model would have to correspond to a level in the free atoms. And the insulating or conducting behavior depends on electron filling of bands. However, when the spin-orbit coupling (SOC) effect is included, this conventional picture is modified with the appearance of an intermediate topological insulator state, as shown in Fig. 1 in the main text and Fig. S1. Here we studied the band evolution of trigonal and honeycomb lattices without and with SOC effect based on the generic tight-binding model discussed in the main text. As shown in Fig. S2, with decreasing average bond length L, the initially separated s and p levels spread to form individual bands. Comparing with the case without SOC effect, an intermediate topological phase appears during the band evolution process when the SOC effect in included.

The above band evolution process is a general trend that works for different situations as long as the band inversion may occur. Therefore, the scaling is applicable for both $\Delta_{sp} \gg \lambda$ [see Fig. S2(a,b)] and $\Delta_{sp} \ll \lambda$ [see Fig. S2(e,f)] cases. For the condition of $\Delta_{sp} \gg \lambda$, the critical transition point γ_c is roughly determined by a critical bandwidth $W_c = \frac{1}{2}(W_s + W_p - 2\lambda) = \Delta_{sp} - \lambda$. For the opposite limit, i.e., $\Delta_{sp} \ll \lambda$, the topological phase transition occurs still at a critical point L_c, but is now determined by the condition: $W_c = \lambda - \Delta_{sp}$.

DETAILS OF METHODOLOGY

Tight-binding model

We consider a general atomic-basis tight-binding model for various crystalline and quasicrystalline lattices with three orbitals (s, p_x, p_y) per site. The Hamiltonian is given by

$$H = \sum_{i\alpha} \epsilon_\alpha c^\dagger_{i\alpha} c_{i\alpha} + \sum_{\langle i\alpha, j\beta \rangle} t_{i\alpha,j\beta} c^\dagger_{i\alpha} c_{j\beta} + i\lambda \sum_i (c^\dagger_{ip_y} \sigma_z c_{ip_x} - c^\dagger_{ip_x} \sigma_z c_{ip_y}), \tag{1}$$

where $c^\dagger_{i\alpha} = (c^\dagger_{i\alpha\uparrow}, c^\dagger_{i\alpha\downarrow})$ and $c_{i\alpha} = (c_{i\alpha\uparrow}, c_{i\alpha\downarrow})^T$ are electron creation and annihilation operators on the $\alpha(= s, p_x, p_y)$ orbital at the i-th site. ϵ_α is the on-site energy of the α orbital. The second term is the hopping term where $t_{i\alpha,j\beta} = t_{\alpha,\beta}(\mathbf{r}_{ij})$ is the hopping integral which depends on the orbital type $(\alpha, \beta = s, p_x, py)$ and the vector $\mathbf{r}_{ij}$ between sites i and j. λ is the SOC strength and σ_z is the Pauli matrix. In our model, the hopping integral $t_{i\alpha,j\beta} = t_{\alpha\beta}(\mathbf{r}_{ij})$ is given by the Slater-Koster parametrization [3]

$$t_{\alpha,\beta}(\mathbf{r}_{ij}) = \mathrm{SK}[\hat{\mathbf{r}}_{ij}, V_{\alpha\beta\delta}(r_{ij})], \tag{2}$$

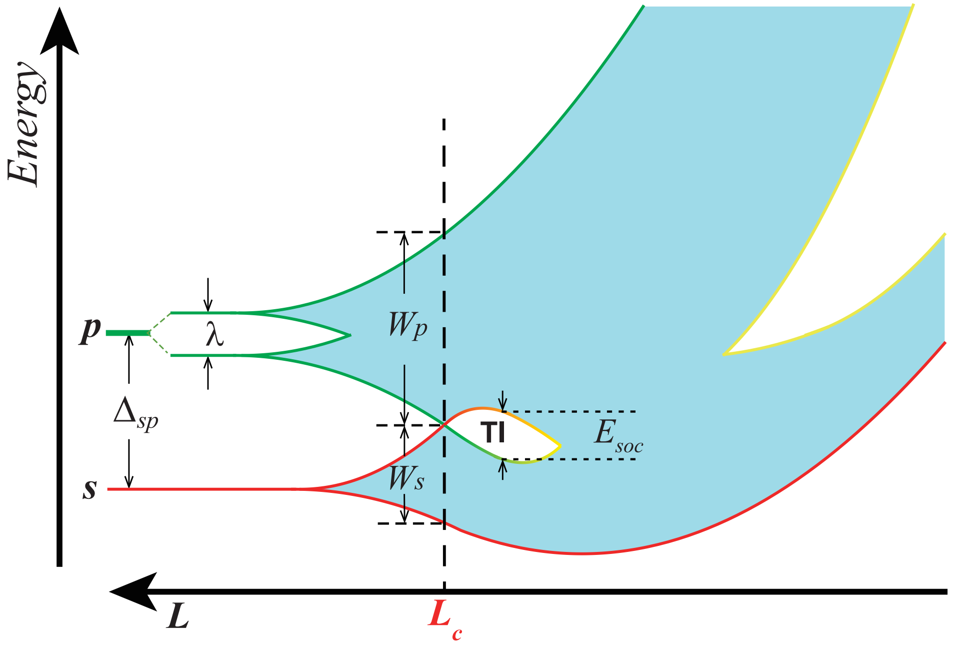

FIG. S1. Schematic illustration of topological phase transition (TPT). By reducing the average bond length L, the bandwidth increases gradually and a TPT occurs at L_c.

where $\hat{\mathbf{r}}_{ij} = (l, m)$ is the unit direction vector. In particular, the formula of SK[$\cdot$] for the s, p_x, p_y orbitals in our model is written as

$$t_{ss} = V_{ss\sigma}, \tag{3}$$
$$t_{sp_x} = lV_{sp\sigma}, \tag{4}$$
$$t_{sp_y} = mV_{sp\sigma}, \tag{5}$$
$$t_{p_xp_x} = l^2V_{pp\sigma} + (1 - l^2)V_{pp\pi}, \tag{6}$$
$$t_{p_yp_y} = m^2V_{pp\sigma} + (1 - m^2)V_{pp\pi}, \tag{7}$$
$$t_{p_xp_y} = lm(V_{pp\sigma} - V_{pp\pi}), \tag{8}$$

where $V_{\alpha\beta\delta} = V_{\alpha\beta\delta}(r_{ij})$ is the $\delta(= \sigma, \pi)$ bonding parameter between $\alpha(= s, p_x, p_y)$ orbital at the i-th site and $\beta(= s, p_x, p_y)$ at j-th site. The distance dependence of the bond integral $V_{\alpha\beta\delta}$ is captured approximately by power-law decay function $V(r_{ij}) \propto \frac{1}{r_{ij}^2}$ or a exponentially decay function $V(r_{ij}) \propto e^{1-r_{ij}}$ in different materials. Since only the band inversion between s and p states of different parities is important for the realization of topological states, we focus mainly on 2/3 filling of electron states hereafter, unless otherwise specified.

We calculate the electronic structures of various periodic lattices using the above tight-binding model. By scanning the average bond length L, we can determine the critical point L_c of energy gap closing for the TPT. We have changed r_{cut} to take different neighboring shells into consideration. Specifically, for periodic lattices in our calculations, we change r_{cut} with different values to consider different cases, such as (i) only first nearest-neighbor (NN) hoppings; (ii) also including second NN hoppings, (iii) third NN hoppings and so on.

To calculate the quasicrystals, we construct quasicrystalline lattice (QL) models according to the Penrose tiling with fivefold rotational symmetry [4, 5] and Ammann-Beenker tiling with eightfold rotational symmetry [6]. Since the QL possesses long-range orientational order but lacks translational symmetry, we cannot use the Bloch theorem as for the crystal calculations. However, it is still possible to generate a series of periodic lattices with a growing number of atoms that approximate the infinite QL according to the quasicrystal tiling approximants [7, 8].

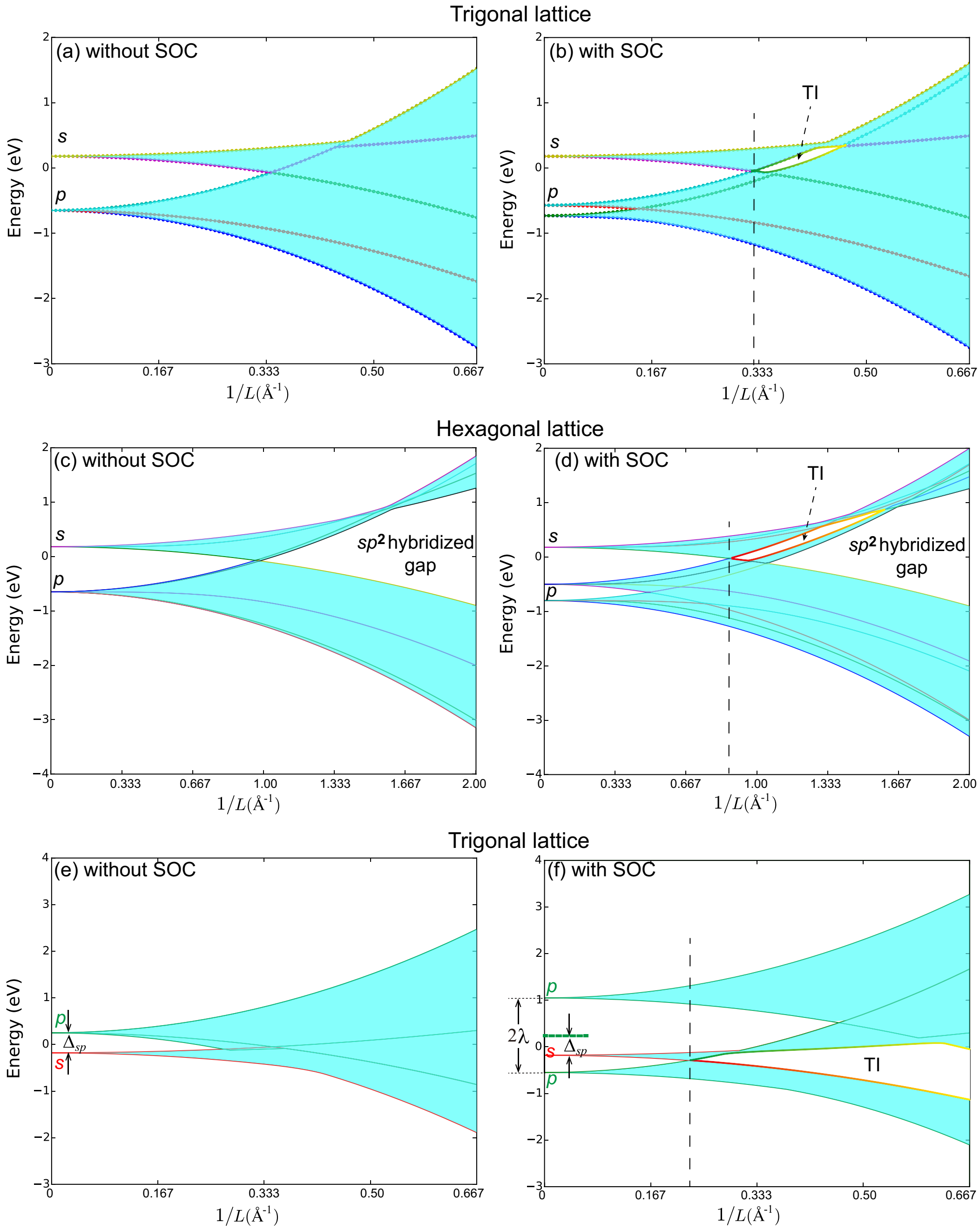

FIG. S2. The calculation of formation of band structure from discrete levels of isolated atoms by increasing the reciprocal of L. (a) In a trigonal lattice without SOC and (b) with SOC. (c) In a hexagonal lattice without SOC and (d) with SOC. In the trigonal lattice, s- and p-levels spread to form s- and p-bands which overlap to form a metallic crystal. In the hexagonal lattice, s- and p-levels spread to form s- and p-bands which overlap in the crystal and reopen a semiconducting gap due to strong sp^2 hybridization. When SOC is included, there are intermediate regions of topological insulator state where band inversion occurs for both lattices. (e,f) The similar calculation as (a, b) in a trigonal lattice for the opposite situation of $\Delta_{sp} = \epsilon_s - \epsilon_p \ll \lambda$.

Calculation of topological invariants

To determine the electronic topology of various periodic lattices with time reversal symmetry, we calculated the $\mathbb{Z}_2$ topological invariant for crystals using the Wannier charge center method as proposed by Soluyanov and Vanderbilt [9]. In the following, we will review the method that enables us to calculate the topological $\mathbb{Z}_2$ invariant of a general time-reversal-symmetric two-dimensional (2D) insulator. In the general case, the $\mathbb{Z}_2$ index is defined by the change of

1D "time-reversal polarization" in the k_y direction, as the other wave vector k_x evolves from 0 to π. The time-reversal polarization can be explicitly visualized by tracing the 1D hybrid Wannier charge centers (WCCs) in the k_y direction as a function of k_x. The 1D hybrid Wannier functions (WFs) localized in the y direction are constructed as

$$|W_{nR_y}(k_x)\rangle = \frac{1}{2\pi}\int_0^{2\pi} dk_y e^{ik_y(r_y - R_y)}|u_{n,\mathbf{k}}\rangle, \tag{9}$$

where R_y is the y component of a lattice vector $\mathbf{R}$ and $|u_{n,\mathbf{k}}\rangle$ is the cell-periodic part of Bloch wavefunction. The hybrid WCC $\langle y_n\rangle(k_x)$ is then defined as the expectation value of $\hat{y}$ for the hybrid WFs in the "home" unit cell $R_y = 0$, i.e., $\langle y_n\rangle(k_x) = \langle W_{n0}|\hat{y}|W_{n0}\rangle$. The $\mathbb{Z}_2$ index is odd if the hybrid WCCs of the Kramers doublets switch partners during the evolution, and even otherwise [10].

In order to verify the topological state in noncrystalline lattices, we adopt another topological invariant, the spin Bott index B_s [4, 5]. In the following, we will review the method that enables us to calculate B_s. First, one construct the projector operator of the occupied states,

$$P = \sum_i^{N_{occ}} |\psi_i\rangle\langle\psi_i|. \tag{10}$$

To make a smooth decomposition $P = P_+ \oplus P_-$ for spin-up and spin-down sectors, one construct the projected spin operator

$$P_z = P\hat{s}_z P, \tag{11}$$

where $\hat{s}_z = \frac{\hbar}{2}\sigma_z$ is the spin operator (σ_z is the Pauli matrix). By solving the eigenvalue problem

$$P_z| \pm \phi_i\rangle = S_\pm| \pm \phi_i\rangle, \tag{12}$$

one can construct new projector operators

$$P_\pm = \sum_i^{N_{occ}/2} | \pm \phi_i\rangle\langle \pm\phi_i|, \tag{13}$$

for two spin sectors. Next, one calculate the projected position operators

$$U_\pm = P_\pm e^{i2\pi X} P_\pm + (I - P_\pm), \tag{14}$$

$$V_\pm = P_\pm e^{i2\pi Y} P_\pm + (I - P_\pm), \tag{15}$$

and the Bott index

$$B_\pm = \frac{1}{2\pi}\mathrm{Im}\{\mathrm{tr}[\log(\tilde{V}_\pm \tilde{U}_\pm \tilde{V}_\pm^\dagger \tilde{U}_\pm^\dagger)]\}, \tag{16}$$

for two spin sectors, respectively. Here X and Y are the rescaled coordinates which are defined in the interval $[0, 1)$. Finally, we define the spin Bott index as the half difference between the Bott indices for the two spin sectors

$$B_s = \frac{1}{2}(B_+ - B_-). \tag{17}$$

TPTS OF TWO-DIMENSIONAL CRYSTALS AND QUASICRYSTALS

To validate the linear scaling of TPT, we calculated the phase diagram with the decreasing average bond length L for different two-dimensional (2D) lattices and determined the critical transition point L_c for the TPT between normal insulators (NIs) and quantum spin Hall (QSH) states (see Fig. S7). Table S1 lists the critical values L_c for typical 2D crystals including oblique, rectangle, rhombic, square, trigonal, honeycomb, Lieb lattices (see Fig. S3), semiregular Archimedean lattices (see Fig. S4) [11], decorated trigonal lattices (see Fig. S5) and quasicrystalline lattices based on Penrose and Ammann-Beenker tilitings (see Fig. S6).

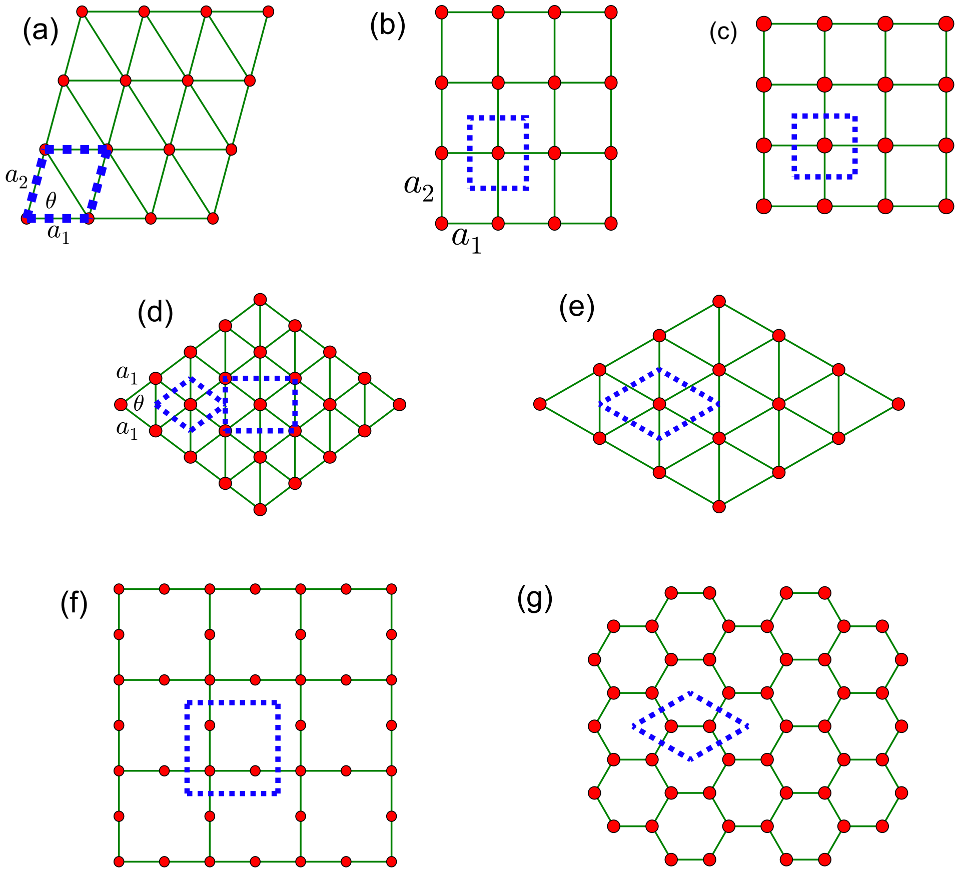

FIG. S3. Five 2D Bravais lattice: (a) oblique, (b) rectangle, (c) square, (d) rhombic or centered rectangle, (e) trigonal and two typical lattices: (f) Lieb, (g) honeycomb lattices. The unit cells are outlined by dashed lines.

RANDOM VACANCY

We simulated the trigonal lattices with random vacancies by constructing a $N \times N$ supercell and randomly removing atoms to create vacancies. $N = 37$ is adopted for the calculation shown in the main text. For each vacancy concentration η, at least 10 random samples are used to obtain the statistical average. As shown in the main text, we calculated the phase diagram in the L-η parameter space for the trigonal lattice with random vacancies. We further checked the size effect by calculating the phase diagram on a larger supercell ($N = 50$). The results show a similar phase diagram including NI, QSH state and gapless phases. And a clear phase boundary between NI and QSH states indicates a TPT which follows the same linear scaling relation discussed in the main text.

THERMAL FLUCTUATION

We adopted the quasi-lattice model to simulate the effect of thermal fluctuation. The basic assumption for the quasi-lattice model is that the atomic displacement ($\mathbf{u}$) away from their equilibrium position follows a Gaussian distribution

$$p(\mathbf{u}) = \frac{1}{\sqrt{2\pi\sigma^2}} \exp(-\frac{u^2}{2\sigma^2}), \tag{18}$$

where the relative mean-squared displacement σ^2 represents the strength of thermal fluctuation which is proportional to temperature T. In such disordered lattices, the Dirac δ function-type coordination number $z_i\delta(r - r_i)$ broadens

TABLE S1. The electron hopping potential γ and the critical bond length L_c for TPT in typical 2D crystals and quasicrystal lattices. ("$1nn$", "$2nn$", and "$3nn$" denote that the calculation includes the first-, second-, and third nearest-neighbor hoppings, respectively.)

Lattices	$1/\gamma$ (Å/eV)	L (Å)	Note
Oblique ($a_1 = 1; a_2 = 0.6; \theta = 70°$)	5.094	7.730	$3nn$
Rectangle ($a_1 = 1; a_2 = 0.5$)	3.891	6.030	$3nn$
Rhombic ($a_1 = 1; \theta = 76°$)	2.146	3.146	$2nn$
Square	1.199	1.681	$1nn$
	3.367	4.971	$2nn$
Trigonal	2.120	3.089	$1nn$
	6.649	9.745	$2nn$
Honeycomb	0.758	1.092	$1nn$
	4.333	6.294	$2nn$
Lieb	1.904	2.787	$2nn$
Snub hexagonal $3^4.6$	1.661	2.339	$1nn$
Enlongated triangular $3^3.4^2$	1.658	2.326	$1nn$
	2.765	4.030	$2nn$
Snub square $3^2.4.3.4$	1.644	2.358	$1nn$
	2.766	4.030	$2nn$
Trihexagonal 3.6.3.6 (Kagome)	1.191	1.747	$1nn$
Small rhombitrihexagonal 3.4.6.4 (Ruby)	2.273	3.186	$2nn$
Truncated square 4.8^2	1.237	1.778	$2nn$
Truncated hexagonal 3.12^2 (Star)	0.801	1.174	$1nn$
Great rhombitrihexagonal 4.6.12	1.239	1.784	$2nn$
	2.400	3.399	$3nn$
Ammann-Beenker quasicrystal	3.384	4.763	$3nn$
Penrose quasicrystal	2.720	3.522	$3nn$

the radial distribution function with increasing σ, which is approximately given by [12–16]

$$g(r) = \frac{r}{\sqrt{2\pi}} \sum_{i=1}^{\infty} \frac{z_i}{r_i \sigma_i} \{\exp[-\frac{(r - r_i)^2}{2\sigma_i^2}] - \exp[-\frac{(r + r_i)^2}{2\sigma_i^2}]\}, \tag{19}$$

where the summation runs over all neighboring shells of the lattice. r_i and z_i are the radius and coordination number of these shells, respectively. Following the general statistical considerations, the dispersions of the Gaussian distributions σ_i^2 obey the law of structural diffusion $\sigma_i^2 \approx \sigma^2 \frac{r_i}{r_1}$ [13, 14].

VALIDITY OF THE LINEAR SCALING FOR DIFFERENT FORM OF ELECTRON HOPPINGS

In order to verify the validity of the universal linear scaling, we studied the TPT in systems with different forms of electron hopping. As an illustration, we also checked the validity of the linear scaling for a different exponentially decay function ($V(r_{ij}) \propto e^{-\alpha(r_{ij}-1)}$). As shown in Fig. S8, the calculated critical points L_c for different lattices follows still a linear scaling albeit with a different slop. This indicates that the linear scaling is applicable for systems with different forms of electron hopping.

[1] W. A. Harrison, *Electronic structure and the properties of solids: the physics of the chemical bond* (Courier Corporation, North Chelmsford, MA, 2012).
[2] O. Madelung, *Introduction to solid-state theory*, Vol. 2 (Springer-Verlag Berlin Heidelberg, 2012).
[3] J. C. Slater and G. F. Koster, Phys. Rev. **94**, 1498 (1954).
[4] H. Huang and F. Liu, Phys. Rev. Lett. **121**, 126401 (2018).

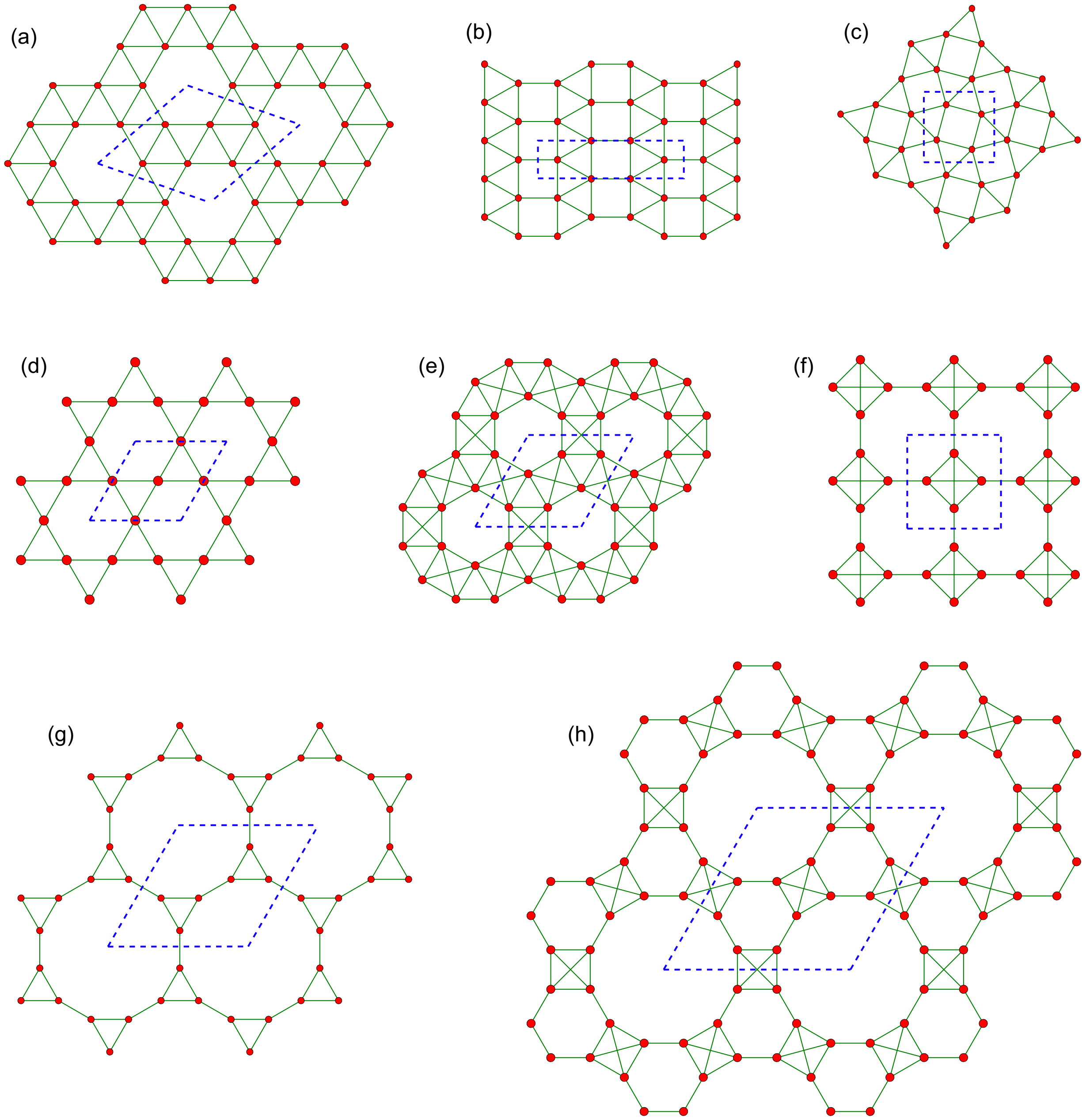

FIG. S4. The eight lattices based on semiregular Archimedean tilings:[11] (a) Snub hexagonal tiling $3^4.6$, (b) elongated triangular tiling $3^3.4^2$, (c) snub square tiling $3^2.4.3.4$, (d) trihexagonal tiling 3.6.3.6 (also known as the Kagome lattice), (e) small rhombitrihexagonal tiling 3.4.6.4 (the Ruby lattice), (f) truncated square tiling 4.8^2, (g) truncated hexagonal tiling 3.12^2 (the Star lattice), and (h) great rhombitrihexagonal tiling 4.6.12. The unit cells are outlined by dashed lines. The critical point of these lattices are presented in Fig.S7 and Fig. S8.

[5] H. Huang and F. Liu, Phys. Rev. B **98**, 125130 (2018).
[6] H. Huang and F. Liu, Phys. Rev. B **100**, 085119 (2019).
[7] H. Tsunetsugu, T. Fujiwara, K. Ueda, and T. Tokihiro, Journal of the Physical Society of Japan **55**, 1420 (1986).
[8] F. P. M. Beenker, *Algebraic theory of non-periodic tilings of the plane by two simple building blocks: a square and a rhombus* (Eindhoven University of Technology, Eindhoven, The Netherlands, 1982).
[9] A. A. Soluyanov and D. Vanderbilt, Phys. Rev. B **83**, 235401 (2011).
[10] C. L. Kane and E. J. Mele, Phys. Rev. Lett. **95**, 146802 (2005).
[11] S. Walter and S. Deloudi, *Crystallography of Quasicrystals: Concepts, Methods and Structures*, Vol. 126 (Springer-Verlag Berlin Heidelberg, 2009).
[12] S. Bagchi, Adv. Phys. **19**, 119 (1970).

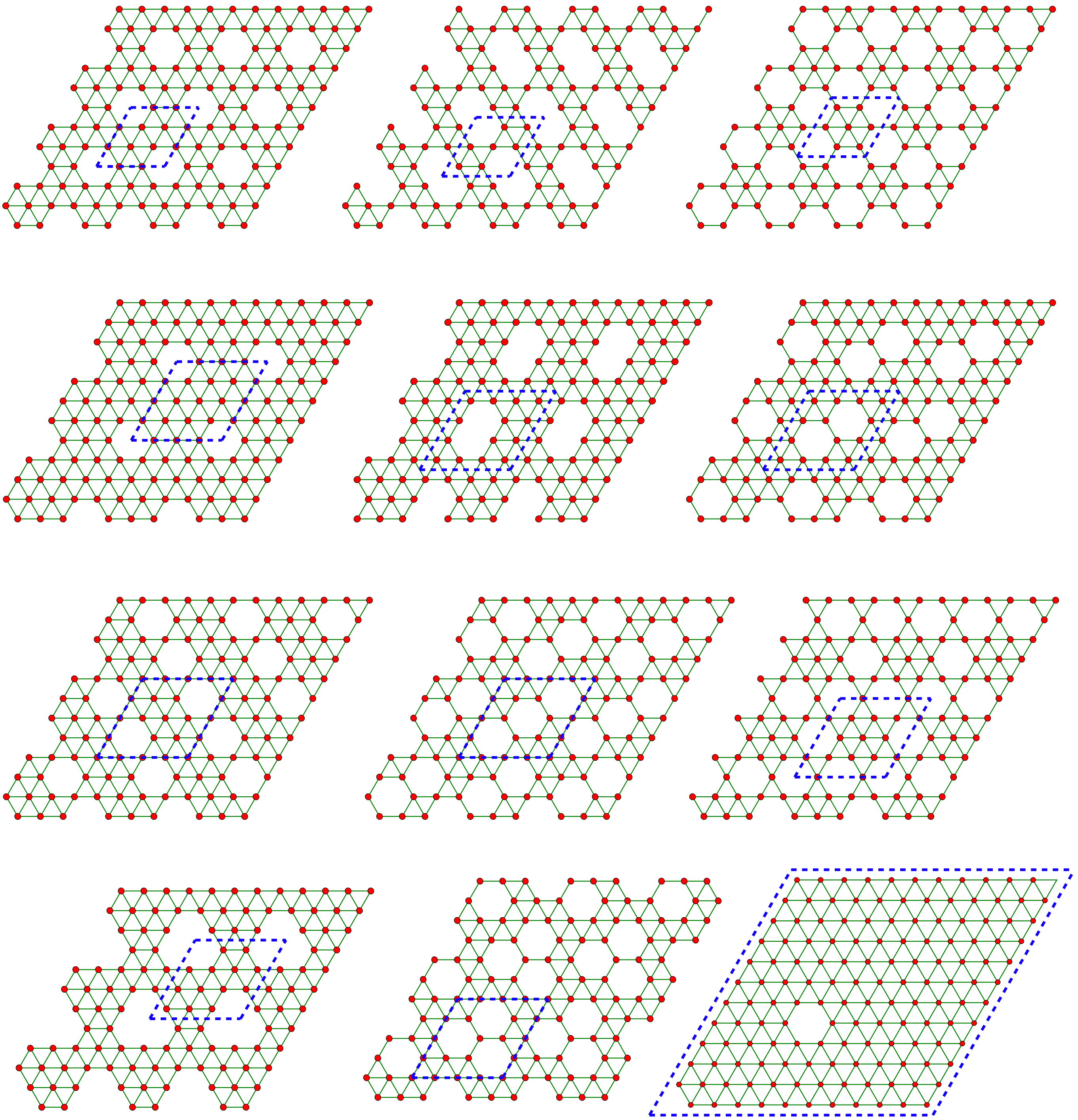

FIG. S5. Several decorated trigonal lattices. The results of these lattices are denoted by open down-pointing grey triangles ("▽") in Fig.S7 and Fig. S8.

[13] S. Franchetti, Il Nuovo Cimento B **10**, 211 (1972).
[14] S. Baer, Physica A **87**, 569 (1977).
[15] N. Medvedev and Y. I. Naberukhin, Phys. Chem. Liq. **6**, 137 (1977).
[16] B. J. Yoon, M. S. Jhon, and H. Eyring, Proc. Natl. Acad. Sci. **78**, 6588 (1981).

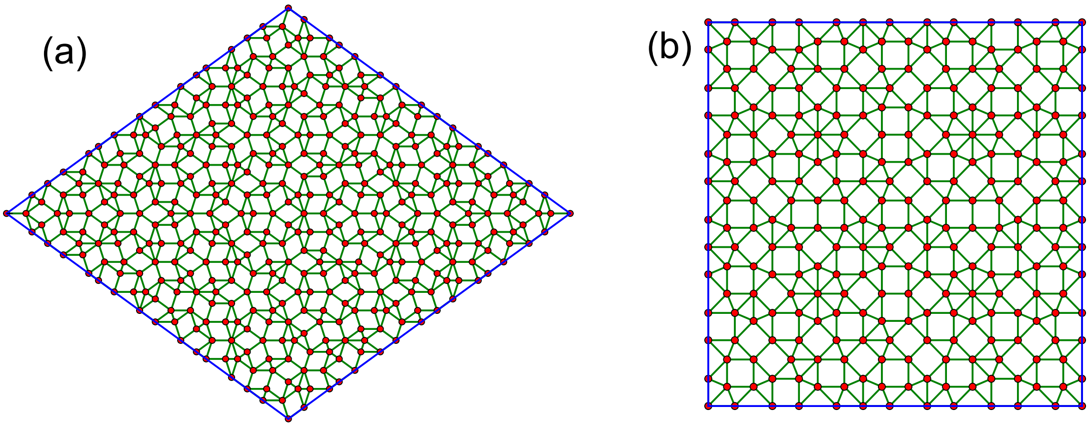

FIG. S6. Atomic model of (a) the Penrose-type and (b) the Ammann-Beenker-type quasicrystal lattices. The blue line defines a unit cell under periodic approximation. In Fig. 3 of the main text, the results of these quasicrystals are denoted by filled orange pentagon ("⬠") and lime octagon ("⯃"), respectively.

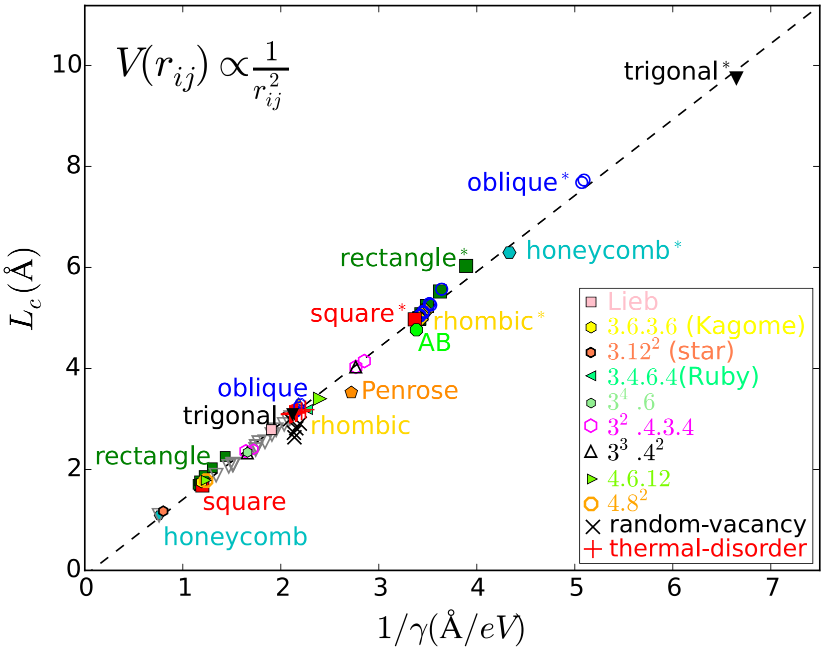

FIG. S7. The linear scaling relation between the critical bond length L_c and the reciprocal of γ for TPT in different 2D crystalline lattices. The power-law-decay function ($\frac{1}{r_{ij}^2}$) for the radial dependence of electron hoppings is used in the calculation. The superscript "*" represents calculations of lattices with a larger r_{cut}.

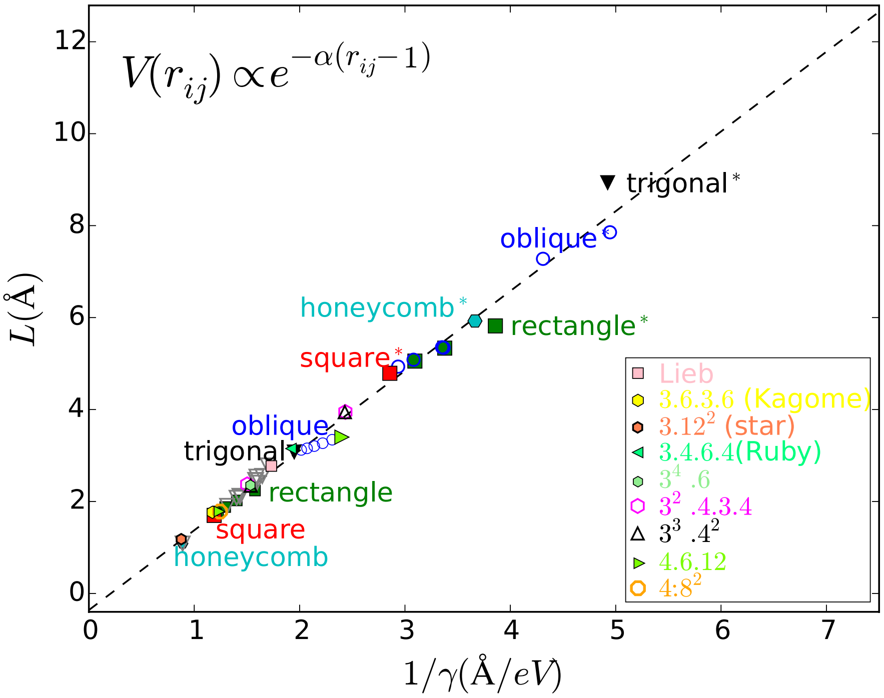

FIG. S8. The linear scaling relation between the critical bond length L_c and the reciprocal of γ for TPT in different 2D crystalline lattices. The exponentially-decay function ($\alpha = 2.3$) for radial dependence of electron hoppings is used in the calculation. The superscript "*" represents calculations of lattices with a larger r_{cut}.
